# Berberine mediates root remodeling in an immature tooth with apical periodontitis by regulating stem cells from apical papilla differentiation

**DOI:** 10.1038/s41368-020-0085-7

**Published:** 2020-06-18

**Authors:** Yujia Cui, Jing Xie, Yujie Fu, Chuwen Li, Liwei Zheng, Dingming Huang, Changchun Zhou, Jianxun Sun, Xuedong Zhou

**Affiliations:** 1 State Key Laboratory of Oral Diseases & National Clinical Center for Oral Diseases & Department of Cariology and Endodontics, West China Hospital of Stomatology, Sichuan University, Chengdu, China; 2 State Key Laboratory of Oral Diseases & National Clinical Center for Oral Diseases & West China Hospital of Stomatology, Sichuan University, Chengdu, China; 3 Shanghai Key Laboratory of Stomatology and Shanghai Research Institute of Stomatology & National Clinical Research Center for Oral Diseases & Department of Oral and Maxillofacial-Head Neck Oncology, Shanghai Ninth People’s Hospital, College of Stomatology, Shanghai Jiao Tong University School of Medicine, Shanghai, China; 4 State Key Laboratory of Oral Diseases & National Clinical Research Center for Oral Diseases & Department of Pediatric Dentistry, West China Hospital of Stomatology, Sichuan University, Chengdu, China; 5 National Engineering Research Center for Biomaterials, Sichuan University, Chengdu, China

**Keywords:** Stem-cell therapies, Mesenchymal stem cells

## Abstract

Once pulp necrosis or apical periodontitis occurs on immature teeth, the weak root and open root apex are challenging to clinicians. Berberine (BBR) is a potential medicine for bone disorders, therefore, we proposed to apply BBR in root canals to enhance root repair in immature teeth. An in vivo model of immature teeth with apical periodontitis was established in rats, and root canals were filled with BBR, calcium hydroxide or sterilized saline for 3 weeks. The shape of the roots was analyzed by micro-computed tomography and histological staining. In vitro, BBR was introduced into stem cells from apical papilla (SCAPs). Osteogenic differentiation of stem cells from apical papilla was investigated by alkaline phosphatase activity, mineralization ability, and gene expression of osteogenic makers. The signaling pathway, which regulated the osteogenesis of SCAPs was evaluated by quantitative real time PCR, Western blot analysis, and immunofluorescence. In rats treated with BBR, more tissue was formed, with longer roots, thicker root walls, and smaller apex diameters. In addition, we found that BBR promoted SCAPs osteogenesis in a time-dependent and concentration-dependent manner. BBR induced the expression of β-catenin and enhanced β-catenin entering into the nucleus, to up-regulate more runt-related nuclear factor 2 downstream. BBR enhanced root repair in immature teeth with apical periodontitis by activating the canonical Wnt/β-catenin pathway in SCAPs.

## Introduction

During the early years, young patient’s teeth are in development. Pulp necrosis and apical periodontitis (AP) as a consequence of trauma or caries arrest root development in injured immature permanent teeth.^1^ This leads to weak root canal walls, open root apexes, and an inadequate crown-root ratio.^2^ It is impossible for these teeth to receive root canal treatment and they are susceptible to fractures.^3^ To preserve the alveolar bone of a growing child, saving immature teeth is critical. The treatment of immature teeth with pulp necrosis and AP remains a challenge.^4^ Currently, the available options are apexification and revascularization.^5^ Apexification can provide an apical barrier against obturation, however, the dentinal walls remain thin^6^ and are prone to root fracture.^7^ In several recent studies, the recurrence of periapical lesions, absence of continued root formation, and intracanal obliteration after revascularization have been reported.^8^ Thus there is an unmet need for a more efficient method of saving immature teeth with AP.

Berberine (BBR) is a benzylisoquinoline alkaloid, an active ingredient in many herbal medicines, including coptis, barberry, and phellodendron.^9^ BBR has been used for diarrhea, dysentery, aphthous stomatitis, and hepatitis.^10,11^ In recent studies, BBR, as a potential medicine for bone disorders, has been of growing interest.^12^ BBR has shown protective effects against bone loss, and restored decreased bone formation in diabetic and postmenopausal osteoporosis.^13–16^ In addition, BBR promoted osteoblast differentiation and new bone area formation.^17^ Moreover, BBR also activated the canonical Wnt/β-catenin pathway in bone marrow mesenchymal stem cells.^18^ In dentistry, BBR has been confirmed to be effective to periodontitis. BBR slowed periodontal degradation through the regulation of matrix metalloproteinases (MMPs).^19^ BBR promoted osteogenic differentiation of human periodontal ligament stem cells (hPDLSCs) by activating the ERK-FOS pathway.^20^

In the present study, we proposed to apply BBR to rat root canals of immature teeth with AP, thereby aiming to enhance root repair. We found that new tissue was observed to significantly form along the roots. To explore the underlying mechanism involved, additional studies were carried out. BBR in different concentrations was introduced into human stem cells from apical papilla (hSCAPs). hSCAPs which resides in the root apex of immature permanent teeth have been related to the formation of odontoblasts for root formation.^21^ A model system that included ex vivo expanded hSCAPs have been shown to regenerate vascularized pulp-like tissue and form dentin-like mineral structures.^22^ Hence hSCAPs were considered to be candidate cells for future regenerative endodontic strategies.^23^ BBR was found to enhance hSCAPs osteogenesis through the canonical Wnt/β-catenin signaling pathway. Together, our data suggested that BBR may be a potential therapeutic agent for root repair in an immature tooth with AP by promoting hSCAPs osteogenesis.

## Results

### BBR enhances tissue repair in immature teeth with AP

**Fig. 1:**
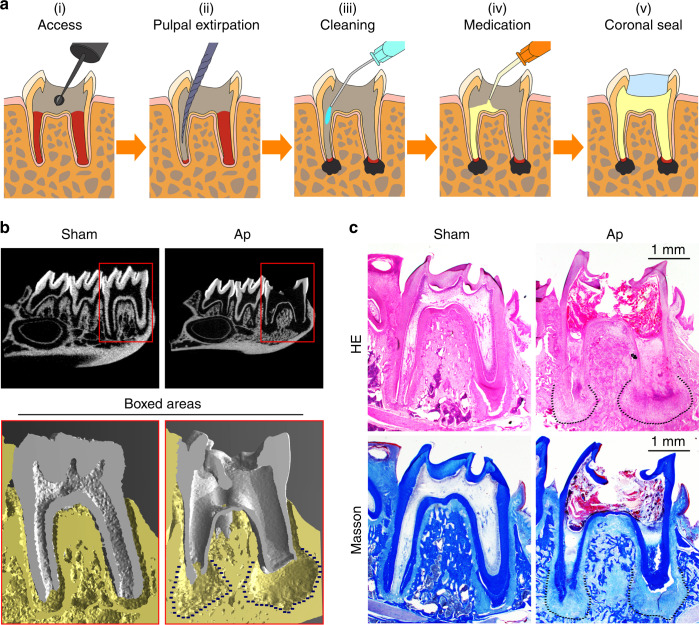
Establishing a model of immature teeth with apical periodontitis. **a** The schematic diagram elucidates the process of model establishment in immature teeth with apical periodontitis. (i) Rats aged four weeks underwent drilling on the center of the occlusal surface. (ii) The pulp was removed from root canals by a #30 K-file and immature teeth with apical periodontitis developed by exposuring the pulp chamber to the oral cavity for 3 weeks. (iii, iv) Root canals were cleaned and treated with berberine (2 mg·mL^−1^), calcium hydroxide, or sterilized saline after removal of the pulp. (v) The coronal access was sealed after treatment. Mandibular samples were collected 3 weeks after coronal sealing. **b** Radiographic results showed that root formation was incomplete and apical periodontitis was established. **c** HE and Masson’s Trichrome staining showed morphological changes at the site of the apical area

**Fig. 2:**
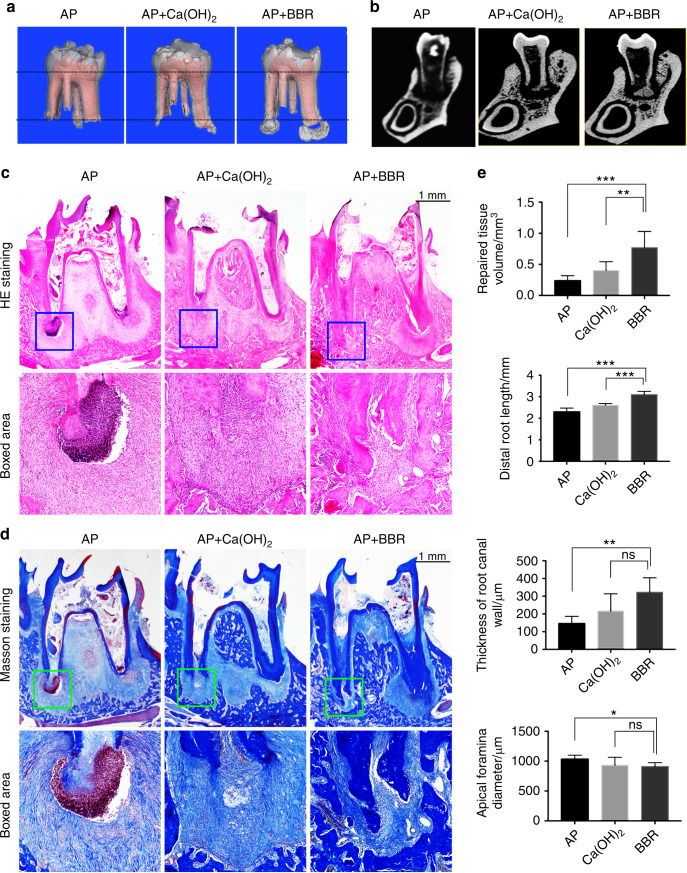
Berberine enhances root repair in immature teeth with apical periodontitis. **a** μ-CT reconstruction showing the shape changes of the tooth and the repaired tissue formation. **b** Representive section diagram from μ-CT showing bone regeneration at the site of apical periodontitis after BBR treatment. **c** HE and **d** Masson staining showing the morphological changes after treatment with BBR. The boxed areas (blue in HE and green in Masson) present detailed information at the site of distal roots in apical periodontitis. **e** Quantitative analysis in μ-CT showing the effect of BBR in rat apical periodontitis model. *P < 0.05, **P < 0.01, ***P < 0.001

After pulp chambers were exposed to the oral cavity for 3 weeks, radiographic and histological results showed that root formation was incomplete and AP was established. The root apexes of the pulp-exposed group were open (Fig. 1b, c). Moreover, the AP group had thinner dentinal walls compared to the control group (Fig. 1b, c). HE and Masson’s trichrome staining showed that inflammation tissue and bone absorption had formed in the AP group (Fig. 1c). The molars were filled with BBR, calcium hydroxide, or sterilized saline for 3 weeks, respectively. The three-dimensional reconstruction of mandibular first molars showed that the distal root length of the Ca(OH)_2_ group and the BBR group increased when compared with the AP group. Furthermore, in the BBR group, significant new tissue had formed in the periapical area (Fig. 2a, b). Radiographic analysis of the distal root involved root length, apical tissue volume, apex diameter, and the volume of apical repaired tissue. The BBR group had a thicker root canal wall and a smaller diameter of the apex compared to AP group, however no significant differences were observed between the BBR group and Ca(OH)_2_ group (Fig. 2e). A significant increase was observed in the volume of the apical repaired tissue and root length in the BBR group when compared with the control group. In addition, the Ca(OH)_2_ group had longer roots compared to the roots in the AP group (Fig. 2e). HE and Masson’s trichrome staining also demonstrated that new tissue had formed along the root apex in the BBR group. Moreover, the AP group showed a large number of inflammatory cells in the periapical area (Fig. 2c, d).

### BBR promotes hSCAPs osteogenesis in a time-dependent and concentration-dependent manner

**Fig. 3:**
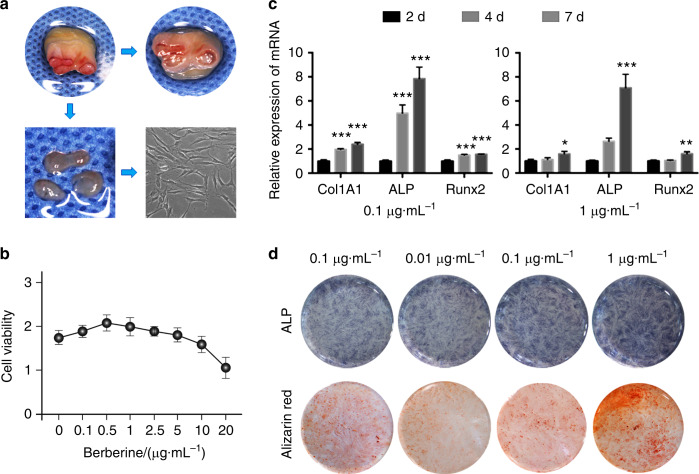
Berberine promotes osteogenesis of human stem cells from apical papilla (hSCAPs) in vitro. **a** Representive images showing hSCAPs isolation from an extracted human third molar. **b** CCK-8 assay showing changes in cell viability using different concentrations of berberine (BBR) in hSCAPs. *Significant difference with respect to the normal control (*P* < 0.05). **c** qPCR showing mRNA expression levels of ColA1, ALP, Runx2 on 2, 4, and 7 days at 0.1 or 1 μg·mL^−1^ BBR. *P < 0.05, **P < 0.01, ***P < 0.001, ****P < 0.000 1. **d** BBR increased the osteogenic capacity of hSCAPs in a dose-dependent manner. ALP staining showing enhanced osteogenesis in hSCAPs by BBR at early stage of osteogenic induction (4 days). Alizarin staining showing the enhanced mineralization capacity in hSCAPs by BBR at the late stage of osteogenic induction (14 days)

To further explore the mechanism of BBR enhancing tissue regeneration in vivo, hSCAPs were separated from the apical papilla of immature third molars (Fig. 3a). BBR stimulated hSCAPs at different concentrations ranging from 0 to 20 μg·mL^−1^. When the concentration was higher than 5 μg·mL^−1^, the cell viability decreased. In addition, a concentration of 20 μg·mL^−1^ BBR showed cytotoxicity. The cell viability stimulated by 1 μg·ml^−1^ BBR was significantly higher than the control (Fig. 3b). Therefore, 0.01, 0.1, and 1 μg·mL^−1^ BBR were introduced into hSCAPs to investigate the osteogenesis of BBR. Our data showed that BBR increased the activity of ALP, a marker for early osteogenesis, which was obvious at higher concentrations (Fig. 3d). The alizarin red staining at 14 days after induction showed the level of mineralization. This revealed that the number of calcified nodules increased in the 0.1 and 1 μg·mL^−1^ BBR group compared to the control group, especially the 1 μg·mL^−1^ group (Fig. 3d). In the 0.1 and 1 μg·mL^−1^ BBR group, the mRNA expression of the osteogenic makers Col1α1 and Runx2 elevated over time (Fig. 3c). These results confirmed that BBR not only promoted osteogenesis but also enhanced osteogenesis in a time-dependent and concentration-dependent manner in hSCAPs.

### BBR stimulates the canonical Wnt/β-catenin signaling pathway to induce osteogenic differentiation of hSCAPs

**Fig. 4:**
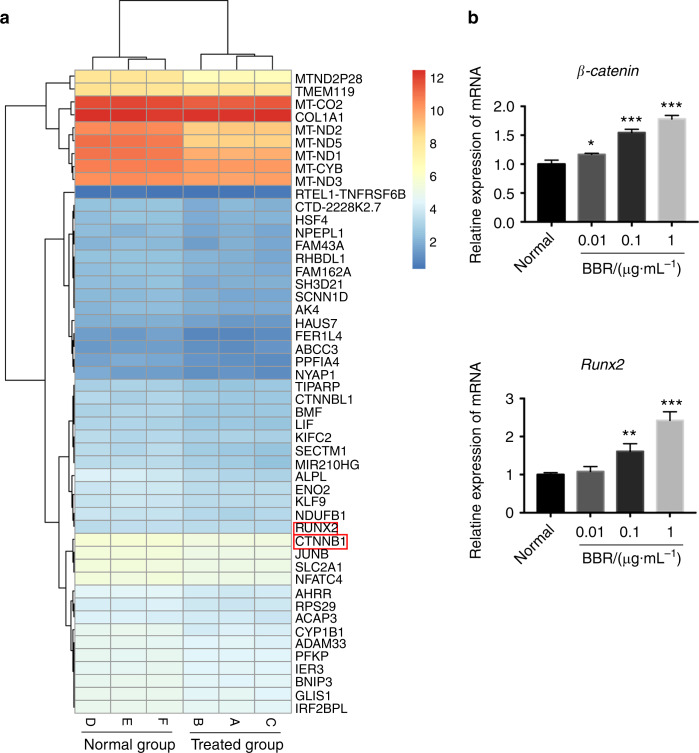
Berberine changes the gene profile involving osteogenesis in hSCAPs. **a** DNA microarray showing changes of the top 50 genes involving osteogenesis in hSCAPs after treatment with berberine (BBR) at 1 μg·mL^−1^. Data are presented as log_2_ (1 + FPKM) and formatted with R software package. FPKM, fragments per kilobase of transcript per million fragments are mapped. **b** Histogram showing the changes in genes β-catenin and Runx2 in hSCAPs induced by BBR at a concentration of 1 μg·mL^−1^ using qRT-PCR. *P < 0.05, **P < 0.01, ***P < 0.001

**Fig. 5:**
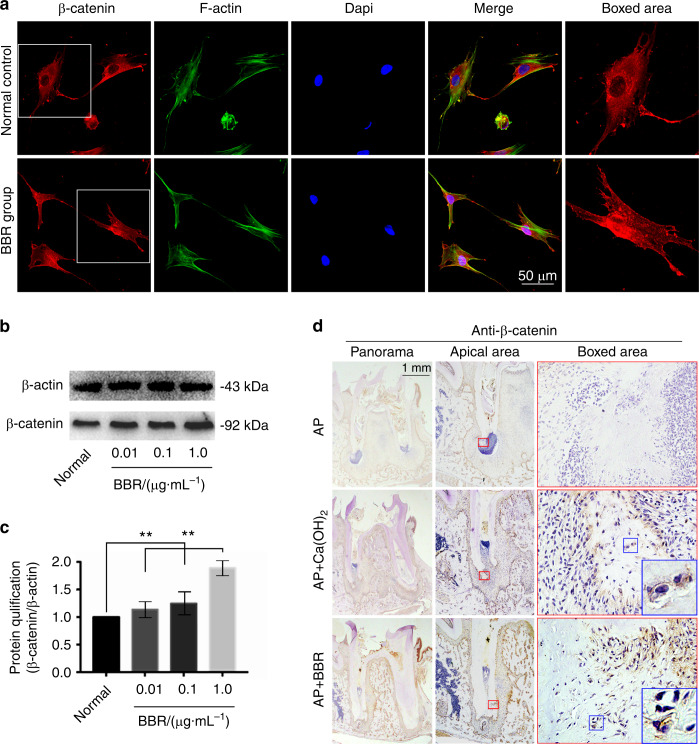
Berberine increases the expression of β-catenin. **a** Representive immunofluorescence staining showing the distribution changes of β-catenin in hSCAPs induced by 1 μg·mL^−1^ berberine (BBR). **b** Western blot analysis showing up-regulation of β-catenin protein in hSCAPs induced by BBR at a concentration of 1 μg·mL^−1^. **c** Quantification in **b** further confirming the increaseing β-catenin in hSCAPs induced by BBR. **Significant difference with respect to the normal control (*P* < 0.01). **d** Representive immunohistochemistry (IHC) staining showing the expression of β-catenin in a rat apical periodontitis model

**Fig. 6:**
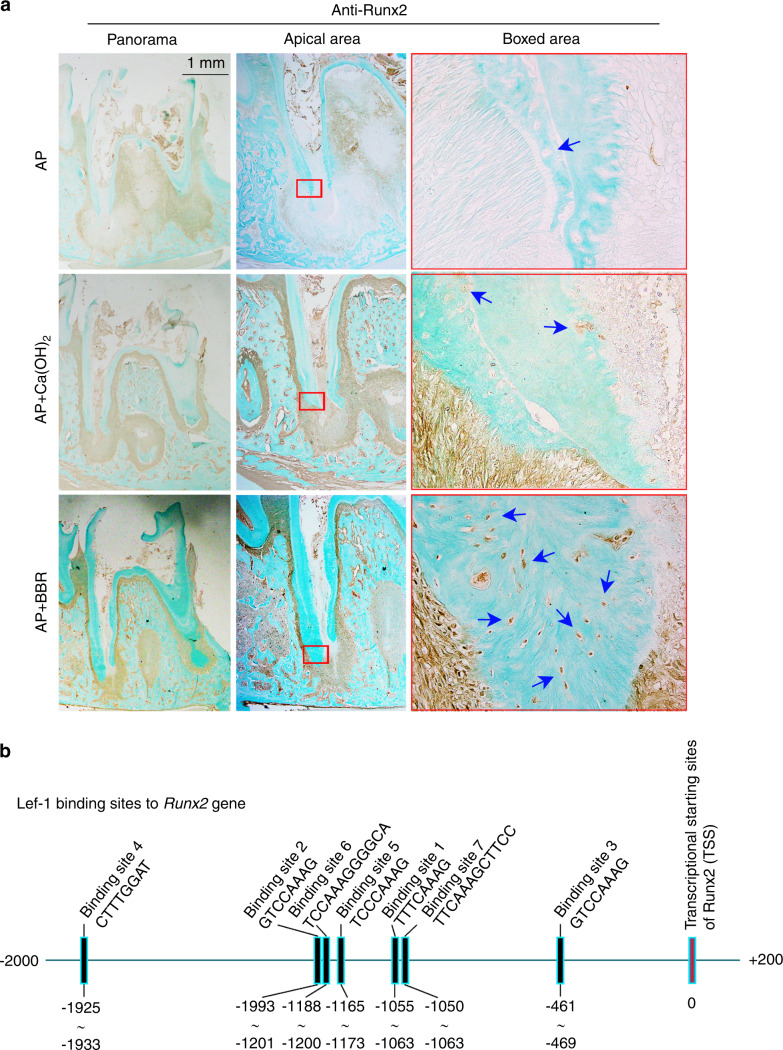
Berberine increases the expression of Runx2. **a** Representive IHC staining showing the expression of Runx2 in the rat apical periodontitis model. **b** Bioinformatic diagram showing potential binding sites of the downstream protein of β-catenin signling, lef-1, in the promoter region of Runx2

To validate involvement of the activated pathway in repaired bone-like tissue, DNA microarray was performed, which showed changes in gene expression. Our data showed that CTNNB1 and Runx2 were upregulated, which indicated involvement in the canonical Wnt/β-catenin-signaling pathway (Fig. 4a). To confirm these findings, qPCR analysis was performed. After incubation with 0.01, 0.1, and 1 μg·mL^−1^ BBR, qRT-PCR results revealed that the expression of β-catenin was significantly increased in a dose-dependent manner (Fig. 4b). Moreover, Runx2, its downstream gene, was also upregulated in a dose-dependent manner (Fig. 4b). Western blot analysis indicated that the total protein of β-catenin incubated with 1 μg·mL^−1^ was higher than that of the control (Fig. 5b). Furthermore, immunofluorescence staining showed that β-catenin was located in both the cytoplasm and nucleus in the 1 μg·mL^−1^ BBR group, whereas it was only present in the cytoplasm in the control group (Fig. 5a). BBR stimulated β-catenin to enter the nucleus. Similarly, the immunohistochemistry results revealed that the number of β-catenin+ cells in the newly formed tissue at the the distal root apex was larger in the BBR group compared to the Ca(OH)_2_ group, however, there was no β-catenin+ cell in the saline group (Fig. 5d). Furthermore, we observed that Runx2 was expressed in the nucleus of bone-like cells in the newly formed tissue, but not in the Ca(OH)_2_ group and the saline group (Fig. 6a). Overall, these results showed that BBR may activate the canonical Wnt/β-catenin-signaling pathway, subsequently induced osteogenic differentiation in hSCAPs, and ultimately repaired root in immature teeth with AP.

## Discussion

The current study showed that BBR enhanced root repair in immature teeth with AP. Based on a rat model of an immature teeth with AP, we applied BBR directly in root canals. We found that BBR significantly promoted tissue formation along the root apex. Moreover, we treated hSCAPs with BBR at different concentrations and found it promoted hSCAPs osteogenesis. To further explore the underlying mechanism involved, we found that BBR stimulated the canonical Wnt/β-catenin-signaling pathway, which resulted in osteogenic differentiation of hSCAPs.

An accumulation of in vitro and in vivo experimental work revealed that BBR may be beneficial to the prevention of musculoskeletal disorders, such as osteoporosis, osteoarthritis, and rheumatoid arthritis, which relied on its ability to target multiple signaling pathways including PKA, p38 MAPK, Wnt/β-catenin, AMPK, RANK/RANKL/OPG, PI3K/Akt, NFAT, NF-κB, Hedgehog, and oxidative stress signaling.^12^ In our study, we found that in the BBR-treated group, root repair was significantly enhanced after BBR had filled root canals for 3 weeks. Radiographic analysis, including root length, apical tissue volume, apex diameter, and thickness of the root wall showed that the BBR group had a greater advantage than the Ca(OH)_2_ group, and the negative control group. However, the repaired tissues were likely ectopic cementum-like and bone-like tissues. Perhaps this was associated with long-time exposure to the inflammatory environment.^24^ It was previously confirmed that high doses of pro-inflammatory cytokines inhibited the efficacy of osteogenic factors.^25^ Furthermore, residual bacteria also downregulated dentinogenic genes^26^ and the amount of mineralized tissue.^27^

We next tried to explore the mechanism of BBR enhancing root repair. Dental papilla are present at the apex of immature teeth, thereby separated from dental pulp by an apical cell-rich zone.^28,29^ Stem cells from apical papilla showed significantly high mineralization and proliferation.^30,31^ Even in an immature tooth with pulp necrosis and AP, human apical papilla retained SCAP vitality.^32^ Therefore, we selected hSCAPs to probe into the mechanism of root repair. In our study, we found that BBR promoted hSCAPs osteogenesis with increased ALP activity and calcified nodules. In addition, the expression of osteogenic makers increased over time. These findings were in line with data published previously on the effect of BBR on other mesenchymal stem cells. BBR accelerated the osteogenesis of periodontal ligament stem cells.^20^ In bone marrow mesenchymal stem cells, BBR stimulated osteogenic differentiation.^18^

Furthermore, the canonical Wnt/β-catenin pathway was dynamically active in tooth-forming regions at every stage of tooth development, and played multiple roles in these events.^33^ Besides, the canonical Wnt/β-catenin promoted proliferation and odonto/osteogenic differentiation in SCAP significantly.^34^ We found that BBR promoted osteogenesis by activating the canonical Wnt/β-catenin signaling pathway. Among targets which were upregulated in DNA microarray, we identified genes CTNNB1 and Runx2, which mark the canonical Wnt/β-catenin-signaling pathway. Increased expression of β-catenin and Runx2 was confirmed by qRT-PCR analysis. These results were in line with the findings of other studies,^35^ in which the canonical Wnt pathway was shown to play an important role in hSCAP differentiation^36^ and tooth formation.^37,38^ In vivo, the IHC results showed that the expression of β-catenin and Runx2 in newly formed tissue were increased in the BBR group. In vitro, BBR upregulated the expression of β-catenin and enhanced β-catenin entering the nucleus to activate downstream gene expression related to osteogenesis. The cytoplasmic protein β-catenin is the key switch in the canonical Wnt pathway.^39,40^ After binding of Wnt to its receptors and after the destruction complex falls apart, β-catenin first accumulates in the cytoplasm, then translocates to the nucleus, and binds transcription factors of the high-mobility-group (HMG) box Tcf/Lef family.^41^ Lef1 binds a site in the promoter of Runx2,^42^ thereby promoting odontogenic differentiation of hSCAPs. Apart from the Wnt/β-catenin-signaling pathway, BBR stimulating osteogenic differentiation may involve other signaling pathways, such as MAPK pathway.^43^

In summary, our study preliminarily demonstrated that BBR stimulated the canonical Wnt/β-catenin-signaling pathway by increasing the expression of β-catenin and enhancing β-catenin entering nuclear, then activated osteogenic genes downstream, and promoted the odontogenic differentiation of hSCAPs. Thus, BBR significantly enhanced root repair. In conclusion, BBR enhanced root repair in an immature tooth with AP by activating the canonical Wnt/β-catenin pathway. Taken together, these findings could potentially save immature teeth with AP.

## Materials and methods

### A model of immature teeth with AP and intracanal medication

All protocols were reviewed and approved by the Institutional Animal Care and Use Committee. All experiments were carried out in accordance with the guidelines and regulations of the Institutional Animal Care and Use Committee. Male wistar rats (4 weeks of age, 70–80 g) were obtained from the Experimental Animal Center of the University. In the mandibular first left molar of 21 rats, access openings were drilled on the center of the occlusal surface to expose the pulp chamber, using a round bur with a diameter of 1 mm. The pulp chamber was exposed to the oral cavity for 3 weeks. As confirmed by radiographs, the establishment of periapical periodontitis was successful. Root canals were cleaned by a #30K file and irrigated by 1% sodium hypochlorite followed by sterile saline. The root canals were dried and filled with 2 mg·mL^−1^ BBR (purity ⩾ 98%, PHR1502, Sigma Aldrich, St. Louis, MO, USA) dissolved in sterile saline, Ca(OH)_2_ (positive control) or sterile saline (negative control). Experimental teeth were sealed with glass ionomer cement (Fig. 1a). Rats were euthanized 3 weeks after treatment. Sealers in the teeth were ensured to be intact, then the jaws were dissected and fixed in 4% paraformaldehyde.

### Micro-computed tomography (μ-CT) analysis

For μ-CT analysis, the mandibles were scanned using μ-CT Scanner (μ-CT50, Scanco, Bassersdorf, Zurich, Switzerland), operated at 70 kV, 200 μA, at an exposure time of 300 ms and a resolution of 10 μm. Repaired tissue of the distal root was uniformly selected under a setting plane at the level of 2 mm underneath the cemento–enamel junction (CEJ). The distal root length was calculated by creating and measuring a straight line from the CEJ to the radiographic apex of the tooth.^44^ The root wall thickness was measured at a point 2 mm along the root length from the CEJ. At this level, the diameter of the apical foramina was measured in buccolingual direction.

### Masson’s trichrome staining

After mandibles were removed from the wax, tissue sections were hydrated by graded alcohol baths and washed using double-distilled water. The Masson’s trichrome staining was conducted according to the manufacturer’s recommendations (Solarbio, G#1340, Beijing, China). The mineralized bone stained blue; intercellular fibers, neuroglia fibers, keratin, and the cytoplasm appeared red; and the nuclei were blue to purple in color. Images were acquired using an inverted light microscopy (Olympus BX53, Tokyo, Japan).

### Hematoxylin and eosin (HE) staining

After dewaxing by xylene and rehydration using graded alcohol baths, sections were stained with HE. Between the hematoxylin staining and eosin staining, sections underwent color separation by HCl–EOTH and ammonia–H_2_O. Images were acquired using an inverted light microscopy (Olympus BX53, Tokyo, Japan).

### Immunohistochemical staining

After deparaffinizing by xylene and hydration using graded alcohol, antigen repair was conducted using citrate antigen retrieval solution (P0081, Beyotime, Shanghai, China) at 100 °C for 15 min. Sections were treated with H_2_O_2_ for 30 min, then blocked for 1 h (β-catenin) or 2 h (Runx2). Diluted primary antibodies (β-catenin:1/100, ab32572 and Runx2:1/400, ab76956, Abcam, Cambridge, UK) were used to incubate the sections at 4 °C overnight. After washing with phosphate-buffered saline (PBS) five times (3 min each time), the tissue slices were incubated with secondary antibody (1:200) for 2 h and horseradish peroxidase for 30 min at room temperature. Proteins were visualized by the DAB solution kit (Vectorlabs, CA, USA).

### Cell culture

Human stem cells of apical papilla (hSCAPs) were isolated from immature third molars without caries, which were extracted from 16 to 18 years old patients. hSCAPs were cultured in minimum essential medium alpha modification (α-MEM, HyClone, Logan, UT, USA) supplemented with 1% penicillin–streptomycin solution, 10% fetal bovine serum (FBS, Gibco, CA, USA). hSCAPs proliferated in plates at 37 °C in 5% CO_2_ until the third passage. To induce hSCAPs osteogenesis, third passage cells were trypsinized and replated in 24-well plates with osteogenic media (10 mM β-glycerophosphate, 50 μmol·L^−1^ ascorbic acid, and 100 nmol·L^−1^ dexamethasone) at a density of 2 × 10^4^ cells per well. As 1 000x stock solutions, BBR chloride (PHR1502, Sigma Aldrich, St. Louis, MO, USA) was dissolved in dimethyl sulfoxide (DMSO) at a concentration of 1 mg·mL^−1^. Then, BBR was diluted to 0.01, 0.1, and 1 μg·mL^−1^ and added to the culture medium, which was changed every 2 days.

### Cell cytotoxicity assay

Cell cytotoxicity was assessed by cell counting kit-8 (CCK-8, Dojindo, Kumamoto, Japan). A total of 5 000 cells per well was seeded in 96-well plates. After 24 h, different concentrations of BBR ranging between 0 and 20 μg·mL^−1^ was added into plate. Plates were incubated for 48 h and CCK-8 solution was added. To calculate the cell viability, the absorbance of each well was measured by reading the optical density at 450 nm.

### Alkaline phosphatase and alizarin red staining

hSCAPs were cultured in osteogenic media for 4 or 14 days and fixed with 4% paraformaldehyde.^45^ The ALP staining kit (Byotime Biotech, Shanghai, China) and alizarin red staining kit (Cyagen, CA, USA) were used in line with the manufacturers’ instructions.

### DNA microarray

For DNA microarrays, hSCAPs were cultured in 1 μg·mL^−1^ BBR or DMSO for 24 h. Based on different hSCAPs from immature third molars of different patients, three independent repeats were performed. Cells were collected by Trizol reagent (no. 15596-026, Thermo Fisher Scientific, MA, USA) and sent for DNA microarray analysis (Shanghai Lifegenes Biotechnology Co., Ltd, Shanghai, China). Before transcriptome sequencing, the RNA quality was checked by using the Bioanalyzer 2100 system (RNA Nano 6000, Agilent Tech, CA, USA). RNA samples were evaluated by the cBot cluster system (HiSeq 4000 PE, CA, USA). For data analysis, HTSeq v0.6.1 was applied for gene reading. Gene expression was described in fragments per kilobase million (FPKM) format. Differentially expressed genes were based on GO and KEGG enrichment analysis.

### Quantitative real-time PCR

Total RNA was extracted by using the RNeasy Plus Mini Kit (Qiagen, CA, USA) according to the manufacturer’s protocol. RNA samples were dissolved in RNase-free water, and quantitated by the Nanodrop spectrophotometer (Nano Spectrophotometer 2000c, Thermo Fisher Scientific, MA, USA). To obtain cDNA, RNA was reverse-transcribed using the cDNA synthesis kit (K1621-RevertAid, Mbi, MD, USA). Quantitative real-time PCR was performed with the SYBR Premix ExTaq II PCR Kit (TAKARA, Shiga, Japan) using an iCycler (Bio-Rad, CA, USA) according to the manufacturer’s protocol. The PCRs contained 1 μmol·L^−1^ for each primer pairs and 1 μL cDNA sample in a 25 μL volume. The PCR program is composed of a 5 s preincubation at 95 °C. Amplification was achieved with 39 cycles of 5 s denaturation at 95 °C, 30 s annealing at 60 °C, and 5 s extension at 72 °C. All experiments were performed in triplicate. Relative expression was calculated using a ^ΔΔ^Ct method by normalizing with gapdh as the internal control.

### Western blot analysis

Cells were cultured for 24 h and treated with BBR or DMSO (control), respectively for 24 h. Cells were washed and lysed in lysis buffer containing protease inhibitor (1% PMSF, Sigma Aldrich, St. Louis, MO, USA). The concentrations of proteins were determined by BCA assay (Beyotime, Shanghai, China). Proteins were separated by SDS/PAGE and transferred to PVDF membranes. Membranes were blocked with 5% non-fat milk for 1 h, and incubated overnight at 4 °C using primary antibodies directed against β-actin, 1:2 000 (sc-47778, ab32572; Abcam, Cambridge, UK); β-catenin, 1:1 000 (ab32572; Abcam, Cambridge, UK), following corresponding secondary antibodies (m-IgGBP-HRP, 1:5 000, sc-516102; mouse anti-rabbit IgG-HRP, 1:5 000, sc-2357, Santa Cruz Biotech, Delaware Avenue, CA, USA) incubated for 2 h at room temperature. β-actin was used as the internal control. Immunocomplexes were visualized using Super Signal reagents (Pierce, Rockford, IL, USA), and ImageJ software (NIH, Bethesda, MD, USA) was used for densitometric analyses of the membranes.

### Immunofluorescence assay

For immunofluorescence analysis, hSCAPs were cultured in 35 mm glass bottom dish for 24 h. A total of 1 μg·mL^−1^ BBR or DMSO was added to the culture medium, and cells were cultured for another 24 h. The culture medium was discarded and samples were washed three times with PBS. Then, cell samples were fixed with 4% cold paraformaldehyde, permeabilized with 0.5% Triton X-100 (Beyotime, Shanghai, China) for 10 min, and blocked with 5% BSA for 1 h. Anti-β-catenin (1:100, ab32572, Abcam, Cambridge, UK) was used to incubate the samples overnight at 4 °C, and a fluorescence-conjugated secondary anti-rabbit antibody (1:100, ab150079, Abcam, Cambridge, UK) incubated for 2 h at room temperature. Nuclei were counterstained with DAPI (D9542, Sigma Aldrich, St. Louis, MO, USA) and phalloidin (A12379, Invitrogen, CA, USA) was applied to stain the cytoskeleton. Confocal images were captured using a confocal microscopy system (Olympus, FV3000, Tokyo, Japan).

### Statistical analysis

Results are presented as the mean ± SEM of at least three individual experiments. Data were analyzed by one-way ANOVA followed by Tukey’s protected least-significant difference post-hoc test for multiple comparisons. *P* < 0.05 was considered statistically significant.
